# *In Vivo* Cannabidiol Treatment Improves Endothelium-Dependent Vasorelaxation in Mesenteric Arteries of Zucker Diabetic Fatty Rats

**DOI:** 10.3389/fphar.2017.00248

**Published:** 2017-05-18

**Authors:** Amanda J. Wheal, Khalid Jadoon, Michael D. Randall, Saoirse E. O’Sullivan

**Affiliations:** ^1^Cardiovascular Research Group, School of Life Sciences, University of Nottingham Medical School, Queen’s Medical CentreNottingham, UK; ^2^School of Medicine, Royal Derby HospitalDerby, UK

**Keywords:** cannabinoid, sodium nitroprusside, vasorelaxation, nitric oxide, cyclooxygenase, ZDF rats

## Abstract

**Background and purpose:** We have shown that *in vitro* treatment with cannabidiol (CBD, 2 h) enhances endothelial function in arteries from Zucker diabetic fatty (ZDF) rats, partly due to a cyclooxygenase (COX)-mediated mechanism. The aim of the present study was to determine whether treatment with CBD *in vivo* would also enhance endothelial function.

**Experimental approach:** Male ZDF rats, or ZDF Lean rats, were treated for 7 days (daily i.p. injection) with either 10mg/kg CBD or vehicle (*n* = 6 per group). Sections of mesenteric resistance arteries, femoral arteries and thoracic aortae were mounted on a wire myograph, and cumulative concentration-response curves to endothelium-dependent (acetylcholine, ACh, 1 nM–100 μM) or endothelium-independent (sodium nitroprusside, SNP, 1 nM–100 μM) agents were constructed. Multiplex analysis was used to measure serum metabolic and cardiovascular biomarkers.

**Key results:** Vasorelaxation to ACh was significantly enhanced in mesenteric arteries from CBD-treated ZDF rats, but not ZDF Lean rats. The enhanced vasorelaxation in ZDF mesenteric arteries was no longer observed after COX inhibition using indomethacin or nitric oxide (NO) inhibition using L-NAME. Increased levels of serum c-peptide, insulin and intracellular adhesion molecule-1 observed in the ZDF compared to ZDF Lean rats were no longer significant after 7 days CBD treatment.

**Conclusion and implications:** Short-term *in vivo* treatment with CBD improves *ex vivo* endothelium-dependent vasorelaxation in mesenteric arteries from ZDF rats due to COX- or NO-mediated mechanisms, and leads to improvements in serum biomarkers.

## Introduction

Cannabidiol is a non-psychoactive phytocannabinoid, which is well tolerated in humans and already on the market as part of a licensed treatment for spasticity in multiple sclerosis (Sativex^®^ GW Pharmaceuticals, Cambridge, UK). CBD alone (Epidiolex^®^, GW Pharmaceuticals, Cambridge, UK) is in Phase 3 clinical trials in children with intractable epilepsies (Dravet Syndrome and Lennox-Gastaut Syndrome). Epidiolex has also received orphan designation status in the United States for the treatment of neonatal hypoxia-ischaemic encephalopathy. CBD is the focus of much research because of its potential in a number of other therapeutic areas. This is due to its anti-inflammatory, anti-convulsant, anti-oxidant, anxiolytic, anti-nausea, anti-tumoural and anti-psychotic properties (Mechoulam et al., 2002; Campos et al., 2016; Fasinu et al., 2016; Leo et al., 2016; Rohleder et al., 2016). A number of preclinical studies have also shown beneficial effects of CBD in a range of disorders of the cardiovascular system (Stanley et al., 2013a).

In diabetes, *in vivo* treatment with CBD is anti-inflammatory and can prevent, and delay, the onset of type 1 diabetes in non-obese diabetic prone mice (Weiss et al., 2006), and decreases myocardial dysfunction, cardiac fibrosis and oxidative stress in diabetic cardiomyopathy (Rajesh et al., 2010). CBD treatment also blocks the increases in iCAM-1 and VEGF in the retina from streptozotocin-induced diabetic rats (El-Remessy et al., 2006). CBD has anti-nociceptive effects against diabetic peripheral neuropathy in mice (Toth et al., 2010) and Sativex is reported to be beneficial in patients with neuropathic pain associated with diabetes or allodynia, although not an official indication for the drug (Hoggart et al., 2015). Together, this suggests that CBD may be effective against a number of diabetes related complications.

Cannabidiol causes vasorelaxation in isolated arteries of rats and in humans (O’Sullivan et al., 2009; Stanley et al., 2015). We have also shown that 2h incubation *in vitro* with CBD can enhance aortic and femoral artery vasorelaxation to ACh (Stanley et al., 2013b). In femoral arteries, this mechanism of endothelium-dependent vasorelaxant enhancement by CBD was mediated via CB_2_ cannabinoid receptors, raised superoxide dismutase activity, elevated COX activity, and activation of vasodilatory EP4 prostanoid receptors (Wheal et al., 2014). Using ZDF rats as a model of type 2 diabetes, the aim of the present study was to determine if chronic *in vivo* treatment with CBD would similarly improve endothelial function across a variety of arteries.

## Materials and Methods

### Animals

Male ZDF rats (ZDF, *n* = 12) and the control strain ZDF Lean rats (Lean, *n* = 12); Charles River, USA) were housed in groups of 2 or 3 in the University of Nottingham Biomedical Services Unit with a 12 h light/dark cycle, a temperature of 22 ± 2°C, and access to Purina 5008 chow and water *ad libitum*. Rats were treated for 7 days (daily i.p. injection) with either 10 mg/kg CBD or vehicle (in a volume of 1 ml/kg; *n* = 6 per group). This dose was chosen based on other positive studies with CBD in diabetic models which have used 5 (Weiss et al., 2008), 10 (El-Remessy et al., 2010; Toth et al., 2010), or 20 (Rajesh et al., 2010; Toth et al., 2010) mg/kg. Pure CBD was dissolved in a vehicle of 3:1:16 solution of ethanol: Tween 80: 0.9% Saline. All procedures were in accordance with the UK Home Office Animal (Scientific Procedures) Act 1986. Following the treatment period, the rats (aged 12–13 weeks old) were stunned by a percussive blow to the head, and killed by cervical dislocation. Post-mortem blood samples were collected, and serum extracted by centrifugation (1000 *g* for 10 min) and then frozen (-80°C) for later use. Blood glucose concentrations were measured using an Accu-Chek Aviva blood glucose monitoring system (Roche Diagnostics Ltd., Mannheim, Germany).

### *Ex Vivo* Assessment of Vasorelaxation

In order to examine the potential effects of CBD on a variety of arteries, we examined small third order arteries of the mesenteric bed (resistance arteries), femoral arteries and the thoracic aorta. Thoracic aortae and femoral arteries were dissected into rings and mounted on fixed hooks, and third-order mesenteric arteries were mounted on 40 μm diameter tungsten wires in a multi-channel wire myograph (Models 610M and 620M, Danish Myo Technology, Aarhus, Denmark). The arteries were bathed in warmed (37°C) and gassed (95% O_2_/5% CO_2_) modified Krebs’–Henseleit solution (mM: 118 NaCl, 4.7 KCl, 1.2 MgSO_4_, 1.2 KH_2_PO_4_, 25 NaHCO_3_, 10 D-glucose, 2 CaCl_2_), with femoral and mesenteric arteries set to a resting tension of 4.9 mN, and thoracic aortic rings set to 9.81 mN. PowerLab 4/30 and 8/30 recording systems were used to record changes in tension (ADInstruments, Oxfordshire, UK). After an equilibration period, the arteries were contracted twice with a high K^+^ ion containing buffer (mM: 62.5 NaCl, 59.4 KCl, 1.2 MgSO_4_, 1.2 KH_2_PO_4_, 25 NaHCO_3_, 10 D-glucose, 2 CaCl_2_) to test for vessel viability. Those arteries which increased in tone above 4.9 mN were considered viable.

Arteries were contracted with methoxamine (α-adrenoceptor agonist (0.5–100 μM), and once a stable tone was reached of approximately 80% of that achieved with high K^+^ buffer, cumulative concentration-response curves to the endothelium-dependent vasorelaxant ACh (1 nM–100 μM) or the NO donor SNP (1 nM–100 μM) were constructed.

In some vessels, the role of NO was assessed by addition of the NO synthase inhibitor L-NAME (300 μM), and the role of prostanoids by was investigated using the COX inhibitor indomethacin (3 μM).

### Serum Biomarkers

The levels of metabolic (Milliplex^®^ MAP Kit RMHMAG-84K) and cardiovascular (Milliplex^®^ MAP Kit RCVD1-89K, Milliplex^®^ MAP Kit RCVD2-89K) biomarkers in serum were quantified using the Luminex^®^ xMAP^®^ technology, using commercially available panels according to the manufacturer’s instructions. Serum levels of ET-1 were measured using a Duoset ELISA kit from R&D systems^®^.

### Data Analysis and Statistical Procedures

Sigmoidal concentration-response curves were fitted by Prism (GraphPad Software, California, USA) to mean percentage relaxations of methoxamine-induced tone, with error bars representing standard error of the mean (SEM), and *n* being the number of arteries from different animals. Maximal relaxation (*R*_max_) and the log of the concentration of agonist that produces a half-maximal response (EC_50_) were calculated from these curves. Comparisons of concentration-response curves in CBD-treated versus vehicle-treated arteries were performed using two-tailed unpaired *t*-tests of calculated EC_50_ and *R*_max_ values, and *P* < 0.05 taken as significant unless otherwise stated. Where sigmoidal curves were not representative of the data, points were joined by lines, and percentage relaxations values at specific concentrations were compared using two-tailed unpaired *t*-test. Results from the serum assays were reported as mean ± SEM, and were compared by 1-way ANOVA with *post hoc* analysis.

### Drugs, Chemical Reagents, and Other Materials

Acetylcholine, methoxamine, SNP, indomethacin, L-NAME and ethanol were purchased from Sigma (Poole, UK). All physiological salts were bought from Fischer Scientific (Loughborough, UK). CBD was a generous gift from GW Research Ltd (Cambridge, UK). 0.9% sodium chloride was made by Macopharma Ltd (Twickenham, UK). TWEEN 80 was produced by Fischer. Stock solutions of indomethacin were made to 10 mM in ethanol, and L-NAME was made to 100 mM in distilled water. Serial dilutions of ACh and SNP were made using distilled water.

## Results

**FIGURE 1:**
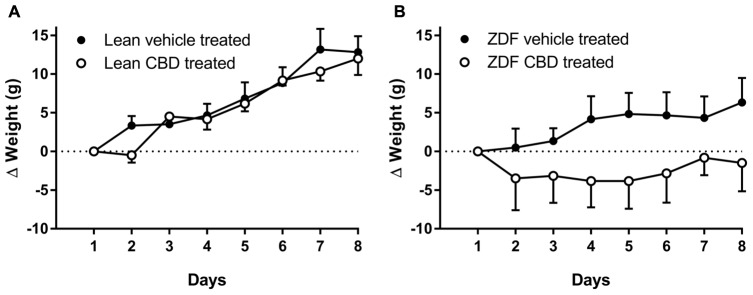
**Change in body weights (Δg from Day 1 weight) of ZDF Lean (A)** and ZDF rats **(B)** recorded daily during 7 days i.p. injection of vehicle or 10mg/kg CBD. On the 8th day, rats were killed by a Schedule 1 procedure. Data are mean ± SD. *n* = 6 animals per group. Data were analyzed by one-way ANOVA and Bonferroni *post hoc* test of selected pairs.

Prior to, and following, 7 days of treatment, ZDF rats were heavier than ZDF lean rats (see **Table 1**). There was no difference in the weight gain over 7 days in the ZDF Lean or diabetic rats treated with CBD compared to vehicle (**Figures 1A,B**). However, the vehicle-treated ZDF rats had a significant increase in weight over time (*P* < 0.05 at day 8) that was not observed in the CBD-treated ZDF rats (see **Figure 1B**). Post-mortem blood glucose levels were not different between groups treated with vehicle versus CBD, but were higher in ZDF rats compared to ZDF Lean rats (*P* < 0.0001, **Table 1**).

**Table 1:** Body weights (g) of ZDF Lean and ZDF rats recorded prior (day 1) and after (day 8) daily i.p. injection of vehicle or 10 mg/kg CBD.

	ZDF vehicle-treated	ZDF CBD-treated	Lean vehicle-treated	Lean CBD-treated
Day 1 Weight (g)	374.0 ± 27.9****	365.0 ± 25.30****	297.3 ± 19.7	288.2 ± 12.4
Day 8 / S1 Weight (g)	380.3 ± 32.0****	363.5 ± 17.00***	310.2 ± 20.0	300.2 ± 13.4
Day 8 post-mortem blood glucose (mM)	20.3 ± 5.8****	23.2 ± 3.3****	7.10 ± 0.77	6.70 ± 1.16

*On the 8th day, rats were reweighed, killed by a Schedule 1 procedure (S1, a percussive blow to the head and cervical dislocation), and their blood glucose levels measured (mM). Data are mean ± SD. *n* = 6 animals per group. Data were analyzed by repeated measures 2 way ANOVA. ^∗∗∗^*P* < 0.001 and ^∗∗∗∗^*P* < 0.0001 denotes a significant difference between ZDF versus lean rats (vehicle-treated versus vehicle-treated, CBD-treated versus CBD-treated).*

### The Effects of CBD Treatment on Vasorelaxant Responses

**FIGURE 2:**
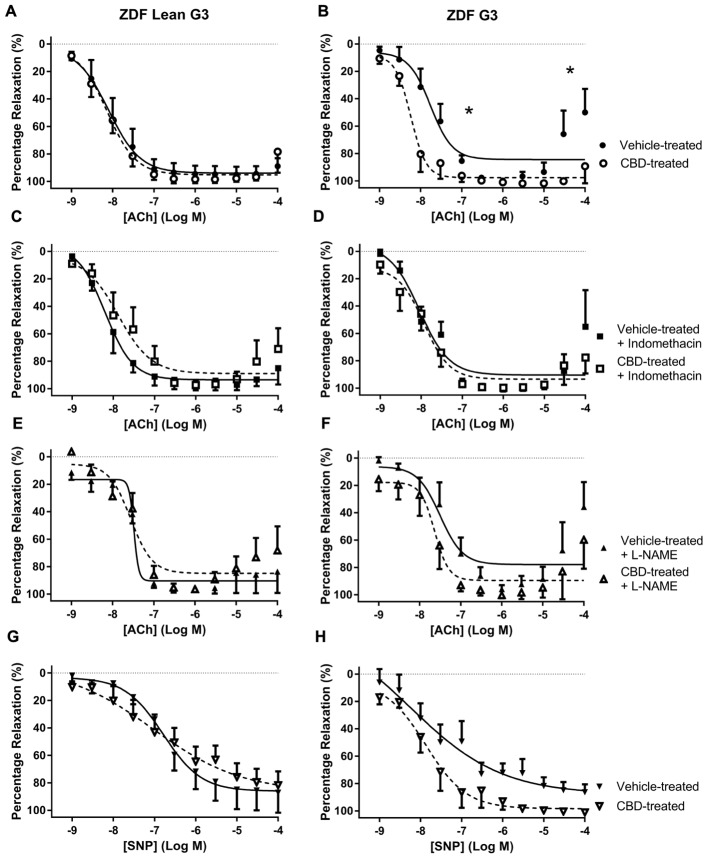
**The effect of CBD (open symbols) or vehicle (closed symbols) treatment on *ex vivo* responses to ACh (A–F)** and SNP **(G,H)** in rings of third-order mesenteric arteries taken from ZDF Lean (left side) or ZDF rats (right side). Some arteries were incubated *in vitro* in the presence of 3 μM indomethacin (squares, **C,D**), or 300 μM L-NAME (upward triangles, **E,F**). Data are mean ± SEM, *n* = 5/6. ^∗^*P* < 0.05 comparing the potency (EC_50_) and efficacy (*R*_max_) of Ach in ZDF arteries after 7 days CBD treatment.

**FIGURE 3:**
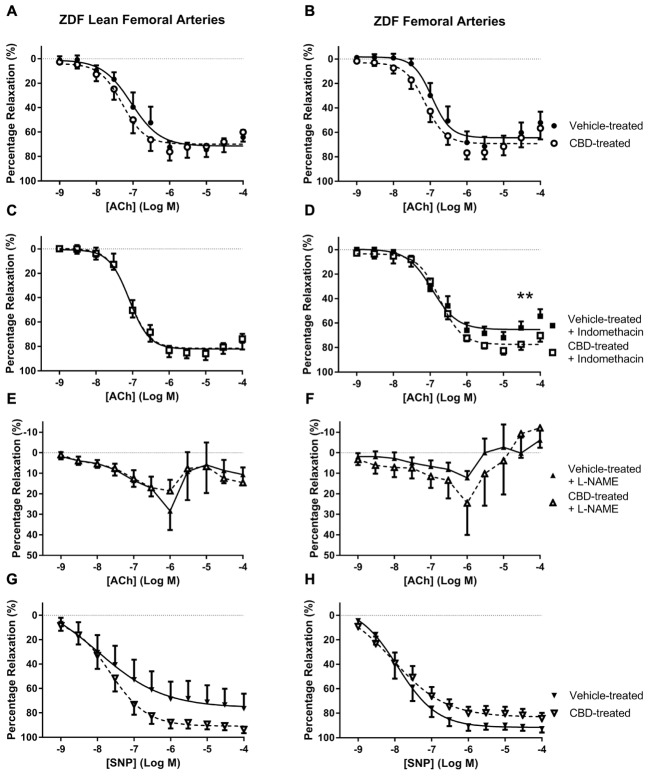
**The effect of CBD (open symbols) or vehicle (closed symbols) treatment on *ex vivo* responses to ACh (A–F)** and SNP **(G,H)** in rings of femoral arteries taken from ZDF Lean (left side) or ZDF rats (right side). Some arteries were incubated *in vitro* in the presence of 3 μM indomethacin (squares, **C,D**), or 300 μM L-NAME (upward triangles, **E,F**). Data are mean ± SEM, *n* = 5/6. ^∗∗^*P* < 0.01 comparing the efficacy (*R*_max_) of Ach in ZDF arteries after 7 days CBD treatment.

**FIGURE 4:**
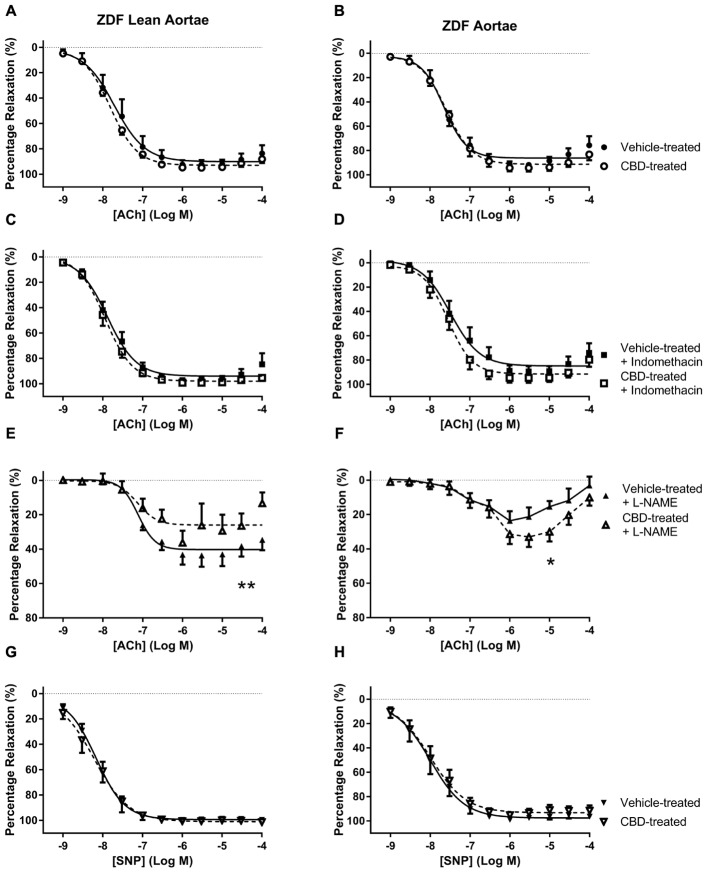
**The effect of CBD (open symbols) or vehicle (closed symbols) treatment on *ex vivo* responses to ACh (A–F)** and SNP **(G,H)** in aortic rings taken from ZDF Lean (left side) or ZDF rats (right side). Some arteries were incubated *in vitro* in the presence of 3 μM indomethacin (squares, **C,D**), or 300 μM L-NAME (upward triangles, **E,F**). Data are mean ± SEM, *n* = 5/6. ^∗^*P* < 0.05 by *t*-test of percentage vasorelaxation at 10 μM Ach comparing responses to Ach in ZDF arteries after 7 days treatment with CBD, ^∗∗^*P* < 0.01 by *t*-test between *R*_max_ values comparing responses to Ach in ZDF arteries after 7 days treatment with CBD.

Treatment with CBD did not alter vasorelaxation to ACh or SNP in any arteries taken from ZDF Lean rats (**Figures 2A, 3A, 4A**). By contrast, treatment of ZDF rats with CBD significantly enhanced the efficacy (EC_50_) and maximal (*R*_max_) response to ACh in third order mesenteric arteries (G3) (**Figure 2B**). The enhanced vasorelaxation to ACh observed in CBD-treated ZDF mesenteric arteries was not seen in the presence of indomethacin (**Figure 2D**) or the NO inhibitor L-NAME (**Figure 2F**). In all vehicle-treated arteries from the ZDF diabetic rats, a contractile response to the highest concentrations of Ach (30 and 100 μM) was observed, but this was not seen in the arteries from ZDF rats that were treated with CBD.

In general, Ach was less efficacious in femoral arteries than G3 (*R*_max_ ∼ 65 versus ∼90% relaxation), especially in the presence of L-NAME, suggesting NO to be the main mediator of vasorelaxation in these arteries. In femoral arteries, an enhanced maximal (*R*_max_) vasorelaxant response to ACh was only observed in CBD-treated ZDF rats in the presence of indomethacin (**Figure 3D**).

In the aortae, treatment with CBD did not change vasorelaxant responses to ACh or SNP in either strain of rat compared to vehicle-treated controls (**Figures 4A,B,G,H**). However, an enhanced vasorelaxation to ACh at 10 μM was seen in aortae from CBD-treated ZDF rats that were pre-incubated with L-NAME (**Figure 4F**). In contrast, in aortae taken from CBD-treated ZDF Lean rats and incubated with L-NAME showed a reduction in the maximal vasorelaxant response to ACh (**Figure 4E**).

### Serum Biomarkers of Metabolic and Cardiovascular Function

**FIGURE 5:**
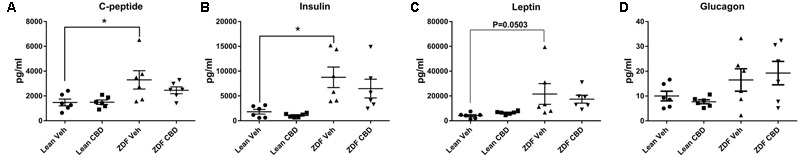
**Circulating metabolic hormones in serum samples from ZDF Lean (circles and squares) and ZDF rats (triangles) that were treated with either vehicle (circles and upward triangles) or 10 mg/kg CBD (i.p., 7 days; squares and downward triangles).** Data are mean ± SEM, with *n* = 6 animals per group. ^∗^*P* < 0.05.

Serum levels of insulin and C-peptide were greater in ZDF rats than ZDF lean rats (*P* < 0.05, **Figures 5A–C**). Seven days treatment with CBD reduced these to a level which was no longer statistically different to the ZDF lean rats. Leptin followed a similar trend, but to a level that did not reach statistical significance (*p* = 0.0503).

There were no significant differences between ZDF lean and ZDF rats, or between vehicle-treated versus CBD-treated rats with regards to serum GLP-1, glucagon, MCP-1, PP, amylin, GIP, IL-6, TNF-alpha or PYY (data not shown).

**FIGURE 6:**
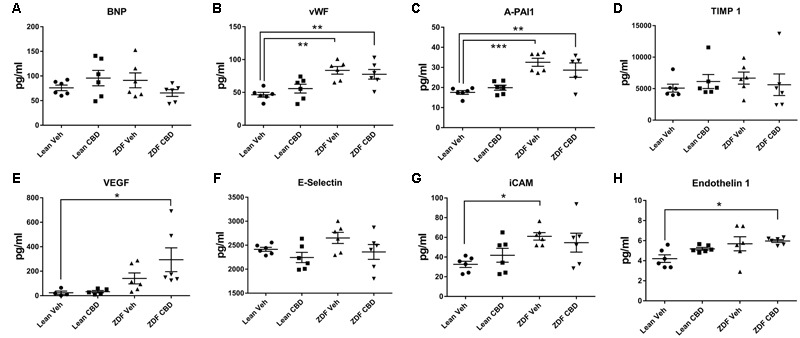
**Circulating cardiovascular biomarkers in serum samples from ZDF Lean (circles and squares) and ZDF rats (triangles) treated with either vehicle (circles and upward triangles) or 10 mg/kg CBD (i.p., 7 days; squares and downward triangles).** Data are mean ± SEM, with *n* = 6 animals per group. ^∗^*P* < 0.05. ^∗∗^*P* < 0.01, ^∗∗∗^*P* < 0.001.

Zucker diabetic fatty rats had higher levels of vWF, A-PAI1, and iCAM than their age-matched ZDF Lean controls (**Figures 6B,C,G**). Treatment of ZDF rats with CBD reduced the level of iCAM to a level that was no longer significantly different to the ZDF lean rats (**Figure 6G**), but levels of vWF and A-PAI1 were not affected. Serum VEGF and endothelin-1 levels were significantly higher in the ZDF rats after CBD-treatment compared to the ZDF Lean controls (**Figures 6E,H**).

## Discussion and Conclusion

The aim of this study was to establish if *in vivo* treatment with CBD could improve vascular function, and we have demonstrated that just 7 days treatment with CBD enhanced endothelium-dependent vasorelaxation in mesenteric arteries from ZDF rats due to COX- and NO-mediated mechanisms. Seven days treatment with CBD was also associated with a positive change in the profile of circulating metabolic and cardiovascular proteins. These data support the growing evidence that cannabinoids, and specifically CBD, maybe be a beneficial treatment of diabetes (Sidney, 2016).

We previously showed that 2 h incubation of arteries *in vitro* with CBD can enhance aortic and femoral artery vasorelaxation to ACh (Stanley et al., 2013b; Wheal et al., 2014). We have now extended this work to show that 7 days treatment *in vivo* with CBD also improves vasorelaxation to ACh. This enhanced vasorelaxant response after CBD treatment was inhibited in the presence of indomethacin or L-NAME, suggesting that this is both COX- and NO-dependent, which is in agreement with our previous findings of COX- (Wheal et al., 2014) and NO- (Stanley et al., 2015) dependent mechanisms of action for CBD in the vasculature. Daily treatment with CBD did not affect vasorelaxation to ACh in mesenteric arteries from the ZDF lean control rats, suggesting that the positive vascular effects of CBD are revealed when vascular dysfunction is present. This is consistent with the known pro-homeostatic properties of cannabinoids, where they only affect the functioning of a perturbed system, but not a healthy one.

Zucker diabetic fatty rats used in this study were heavier, and had higher glucose levels, than the lean control rats. In ZDF diabetic rats, a contractile response to ACh was observed in mesenteric arteries at concentrations above 10 μM that was not observed in the CBD-treated diabetic animals or the ZDF lean control rats (see **Figure 2B**). This contractile response was also partly reduced in the presence of indomethacin (**Figure 2D**), thus it is tempting to suggest that this contractile response in the diabetic rats (not observed in the lean controls) is as a consequence of COX-derived vasoconstrictor production (Feletou et al., 2011; Matsumoto et al., 2015), and that CBD inhibits either the production, or action, of these COX-derived vasoconstrictors in diabetic rats.

In femoral arteries, treatment with CBD did not significantly improve vasorelaxation to ACh or SNP, but there was a trend for enhanced responses in both the lean and diabetic rats, which became significant for diabetic rats in the presence of indomethacin (**Figure 3D**). Similarly, although CBD treatment did not affect vasorelaxation to ACh in diabetic aortae, a significant effect of CBD was revealed in the presence of L-NAME (**Figure 4F**). The ability of CBD to augment endothelium-dependent vasorelaxation when either of the two of the main vasodilatory pathways are inhibited suggests that CBD may be enhancing vasorelaxation through another as yet unidentified mechanism. It also highlights the benefit of CBD in maintaining endothelium-dependent vasorelaxation in diabetic arteries when either vasodilator prostanoids or NO become more dysfunctional (Feletou et al., 2011; Prieto et al., 2014).

The ZDF rats used in this study had significantly raised circulating levels of c-peptide, insulin, leptin, vWF, A-PAI1 and iCAM, as would be expected from a model of type 2 diabetes. Treatment with CBD for 7 days reduced the levels of c-peptide, insulin, leptin and iCAM, such that they were no longer significantly different to the ZDF lean controls. Although these values were not significantly different from the vehicle-treated ZDF diabetic rats, we predict that more prolonged treatment with, or higher dose of, CBD might show a greater effect. Of note, the metabolic parameters were particularly affected by CBD, but without changes in blood glucose levels.

We recently performed a Phase II clinical trial assessing the effects of CBD on HDL-cholesterol in type 2 diabetic patients (Jadoon et al., 2016). A range of cardiovascular secondary and tertiary endpoints were included, although none were significantly affected by CBD, we suspect it was because the dose of CBD was too low. In the present study we used a dose of 10 mg/kg (equivalent to 800 mg per day in an 80 kg person), while in the clinical trial we used a dose of 100 mg, twice daily. In a recent study, we showed that a single dose of 600 mg CBD significantly reduced blood pressure and the blood pressure response to stress in healthy volunteers Please update (Jadoon et al., 2016). Further clinical studies with diabetic patients are required to examine doses of CBD more equitable to those observed in preclinical diabetes studies.

Of the biomarkers tested, circulating levels of VEGF and Endothelin-1 were raised by CBD treatment in the ZDF diabetic rats. We have previously found that CBD significantly increases VEGF secretion from endothelial cells (Hind et al., 2016), while El-Remessey and colleagues showed that CBD reduced retinal expression of VEGF in streptozotocin-treated rats (El-Remessy et al., 2006). We are not aware of any studies that have investigated the effects of CBD on Endothelin-1, although an increase in endothelin-1 is not consistent with an improvement in vascular function. Thus, the vascular significance of changes in VEGF or Endothelin-1 with CBD requires further investigation.

In conclusion, this study has shown that a short *in vivo* treatment protocol with CBD was associated with improvements in endothelium-dependent vasorelaxation in mesenteric arteries, and an improvement in the profile of cardiovascular and metabolic parameters. Further research is required to establish if this effect can be maintained and/or enhanced by more prolonged CBD treatment. The current study supports the growing evidence that CBD may be beneficial against a number of problems associated with diabetes including inflammation (Weiss et al., 2006, 2008; Lehmann et al., 2016), endothelial dysfunction (Rajesh et al., 2007), cardiomyopathy (Rajesh et al., 2010), retinal function (El-Remessy et al., 2006, 2010), and neuropathic pain (Toth et al., 2010).

## Author Contributions

AW: *In vivo* dosing, myography, serum Milliplex assays, experimental design and execution, data analysis and interpretation. Manuscript preparation and revision. KJ: Conducted Multiplex assays. Review of final manuscript. MR: Supervision of AW, experimental design, data analysis and interpretation, and manuscript revision. SO: Supervision of AW and KJ, conducted Multiplex assays, experimental design, data analysis and interpretation, and manuscript preparation and revision. ^∗∗∗^*P* < 0.001.

## Conflict of Interest Statement

The authors declare that the research was conducted in the absence of any commercial or financial relationships that could be construed as a potential conflict of interest.

## Abbreviations

ACh: acetylcholine
APAI1: active plasminogen activator inhibitor-1
BNP: brain natriuretic peptide
CBD: cannabidiol
COX: cyclooxygenase
C-Peptide: connecting peptide
EDHF: endothelium-derived hyperpolarizing factor
ERK1/2: extracellular regulated kinase 1/2
GIP: gastrointestinal peptide
GLP-1: glucagon-like peptide-1
iCAM: intracellular adhesion molecule-1
IL-6: interleukin-6
MCP-1: monocyte chemoattractant protein
NO: nitric oxide
PP: pancreatic polypeptide
PYY: peptide YY
S1: schedule 1 method
SNP: sodium nitroprusside
TIMP 1: tissue inhibitor of metalloproteinases 1
TNFα: tumor necrosis factor alpha
VEGF: vascular endothelial growth factor
vWF: von Willebrand factor
ZDF lean rat: non-diabetic control strain for ZDF rats
ZDF rat: Zucker diabetic fatty rat

## References

[B1] Campos A. C. Fogaca M. V. Sonego A. B. Guimaraes F. S. (2016). Cannabidiol, neuroprotection and neuropsychiatric disorders. *Pharmacol. Res.* 112 119–127. 10.1016/j.phrs.2016.01.033 26845349

[B2] El-Remessy A. B. Al-Shabrawey M. Khalifa Y. Tsai N. T. Caldwell R. B. Liou G. I. (2006). Neuroprotective and blood-retinal barrier-preserving effects of cannabidiol in experimental diabetes. *Am. J. Pathol.* 168 235–244. 10.2353/ajpath.2006.050500 16400026 PMC1592672

[B3] El-Remessy A. B. Khalifa Y. Ola S. Ibrahim A. S. Liou G. I. (2010). Cannabidiol protects retinal neurons by preserving glutamine synthetase activity in diabetes. *Mol. Vis.* 16 1487–1495. 20806080 PMC2925907

[B4] Fasinu P. S. Phillips S. ElSohly M. A. Walker L. A. (2016). Current status and prospects for cannabidiol preparations as new therapeutic agents. *Pharmacotherapy* 36 781–796. 10.1002/phar.1780 27285147

[B5] Feletou M. Huang Y. Vanhoutte P. M. (2011). Endothelium-mediated control of vascular tone: COX-1 and COX-2 products. *Br. J. Pharmacol.* 164 894–912. 10.1111/j.1476-5381.2011.01276.x 21323907 PMC3195913

[B6] Hind W. H. England T. J. O’Sullivan S. E. (2016). Cannabidiol protects an in vitro model of the blood-brain barrier from oxygen-glucose deprivation via PPARgamma and 5-HT1A receptors. *Br. J. Pharmacol.* 173 815–825. 10.1111/bph.13368 26497782 PMC4761095

[B7] Hoggart B. Ratcliffe S. Ehler E. Simpson K. H. Hovorka J. Lejcko J. (2015). A multicentre, open-label, follow-on study to assess the long-term maintenance of effect, tolerance and safety of THC/CBD oromucosal spray in the management of neuropathic pain. *J. Neurol.* 262 27–40. 10.1007/s00415-014-7502-9 25270679

[B8] Jadoon K. A. Ratcliffe S. H. Barrett D. A. Thomas E. L. Stott C. Bell J. D. (2016). Efficacy and safety of cannabidiol and tetrahydrocannabivarin on glycemic and lipid parameters in patients with type 2 diabetes: a randomized, double-blind, placebo-controlled, parallel group pilot study. *Diabetes Care* 39 1777–1786. 10.2337/dc16-0650 27573936

[B9] Lehmann C. Fisher N. B. Tugwell B. Szczesniak A. Kelly M. Zhou J. (2016). Experimental cannabidiol treatment reduces early pancreatic inflammation in type 1 diabetes. *Clin. Hemorheol. Microcirc.* 64 655–662. 10.3233/ch-168021 27767974

[B10] Leo A. Russo E. Elia M. (2016). Cannabidiol and epilepsy: rationale and therapeutic potential. *Pharmacol. Res.* 107 85–92. 10.1016/j.phrs.2016.03.005 26976797

[B11] Matsumoto T. Goulopoulou S. Taguchi K. Tostes R. C. Kobayashi T. (2015). Constrictor prostanoids and uridine adenosine tetraphosphate: vascular mediators and therapeutic targets in hypertension and diabetes. *Br. J. Pharmacol.* 172 3980–4001. 10.1111/bph.13205 26031319 PMC4543607

[B12] Mechoulam R. Parker L. A. Gallily R. (2002). Cannabidiol: an overview of some pharmacological aspects. *J. Clin. Pharmacol.* 42 11S–19S. 10.1002/j.1552-4604.2002.tb05998.x 12412831

[B13] O’Sullivan S. E. Sun Y. Bennett A. J. Randall M. D. Kendall D. A. (2009). Time-dependent vascular actions of cannabidiol in the rat aorta. *Eur. J. Pharmacol.* 612 61–68. 10.1016/j.ejphar.2009.03.010 19285060

[B14] Prieto D. Contreras C. Sanchez A. (2014). Endothelial dysfunction, obesity and insulin resistance. *Curr. Vasc. Pharmacol.* 12 412–426. 10.2174/1570161112666140423221008 24846231

[B15] Rajesh M. Mukhopadhyay P. Batkai S. Hasko G. Liaudet L. Drel R. (2007). Cannabidiol attenuates high glucose-induced endothelial cell inflammatory response and barrier disruption. *Am. J. Physiol. Heart Circ. Physiol.* 293 H610–H619. 10.1152/ajpheart.00236.2007 17384130 PMC2228254

[B16] Rajesh M. Mukhopadhyay P. Batkai S. Patel V. Saito K. Matsumoto S. (2010). Cannabidiol attenuates cardiac dysfunction, oxidative stress, fibrosis, and inflammatory and cell death signaling pathways in diabetic cardiomyopathy. *J. Am. Coll. Cardiol.* 56 2115–2125. 10.1016/j.jacc.2010.07.033 21144973 PMC3026637

[B17] Rohleder C. Muller J. K. Lange B. Leweke F. M. (2016). Cannabidiol as a potential new type of an antipsychotic. A critical review of the evidence. *Front. Pharmacol.* 7:422 10.3389/fphar.2016.00422 PMC5099166 27877130

[B18] Sidney S. (2016). Marijuana use and type 2 diabetes mellitus: a review. *Curr. Diab. Rep.* 16 117 10.1007/s11892-016-0795-6 27747490

[B19] Stanley C. P. Hind W. H. O’Sullivan S. E. (2013a). Is the cardiovascular system a therapeutic target for cannabidiol? *Br. J. Clin. Pharmacol.* 75 313–322. 10.1111/j.1365-2125.2012.04351.x 22670794 PMC3579247

[B20] Stanley C. P. Hind W. H. Tufarelli C. O’Sullivan S. E. (2015). Cannabidiol causes endothelium-dependent vasorelaxation of human mesenteric arteries via CB1 activation. *Cardiovasc. Res.* 107 568–578. 10.1093/cvr/cvv179 26092099 PMC4540144

[B21] Stanley C. P. Wheal A. J. Randall M. D. O’Sullivan S. E. (2013b). Cannabinoids alter endothelial function in the Zucker rat model of type 2 diabetes. *Eur. J. Pharmacol.* 720 376–382. 10.1016/j.ejphar.2013.10.002 24120371

[B22] Toth C. C. Jedrzejewski N. M. Ellis C. L. Frey W. H. II. (2010). Cannabinoid-mediated modulation of neuropathic pain and microglial accumulation in a model of murine type I diabetic peripheral neuropathic pain. *Mol. Pain* 6:16 10.1186/1744-8069-6-16 PMC2845559 20236533

[B23] Weiss L. Zeira M. Reich S. Har-Noy M. Mechoulam R. Slavin S. (2006). Cannabidiol lowers incidence of diabetes in non-obese diabetic mice. *Autoimmunity* 39 143–151. 10.1080/08916930500356674 16698671

[B24] Weiss L. Zeira M. Reich S. Slavin S. Raz I. Mechoulam R. (2008). Cannabidiol arrests onset of autoimmune diabetes in NOD mice. *Neuropharmacology* 54 244–249. 10.1016/j.neuropharm.2007.06.029 17714746 PMC2270485

[B25] Wheal A. J. Cipriano M. Fowler C. J. Randall M. D. O’Sullivan S. E. (2014). Cannabidiol improves vasorelaxation in Zucker diabetic fatty rats through cyclooxygenase activation. *J. Pharmacol. Exp. Ther.* 351 457–466. 10.1124/jpet.114.217125 25212218

